# Inflammatory predisposition predicts disease phenotypes in muscular dystrophy

**DOI:** 10.1186/s41232-016-0019-0

**Published:** 2016-09-05

**Authors:** Yuko Nitahara-Kasahara, Shin’ichi Takeda, Takashi Okada

**Affiliations:** 1 Department of Biochemistry and Molecular Biology, Nippon Medical School, Bunkyo-ku Tokyo, Japan; 2 Department of Molecular Therapy, National Institute of Neuroscience, National Center of Neurology and Psychiatry, Kodaira Tokyo, Japan

**Keywords:** IL-10, Anti-inflammation, Muscular dystrophy, Animal model

## Abstract

Duchenne muscular dystrophy is an incurable genetic disease that presents with skeletal muscle weakness and chronic inflammation and is associated with early mortality. Indeed, immune cell infiltration into the skeletal muscle is a notable feature of the disease pathophysiology and is strongly associated with disease severity. Interleukin (IL)-10 regulates inflammatory immune responses by reducing both M1 macrophage activation and the production of pro-inflammatory cytokines, thereby promoting the activation of the M2 macrophage phenotype. We previously reported that genetic ablation of IL-10 in dystrophic mice resulted in more severe phenotypes, in regard to heart and respiratory function, as evidenced by increased macrophage infiltration, high levels of inflammatory factors in the muscle, and progressive cardiorespiratory dysfunction. These data therefore indicate that IL-10 comprises an essential immune-modulator within dystrophic muscles. In this review, we highlight the pivotal role of the immune system in the pathogenesis of muscular dystrophy and discuss how an increased understanding of the pathogenesis of this disease may lead to novel therapeutic strategies.

## Background

Muscular disorders are a heterogeneous group of genetic diseases caused by mutations in genes encoding sarcolemmal, sarcomeric, and cytosolic muscle proteins. Deficiency or loss of function in any of these proteins leads to varying degrees of progressive loss of motor control. In particular, contraction of dystrophin-deficient myofibers produces severe damage and generates cycles of muscle fiber necrosis and regeneration.

In muscular dystrophy, dystrophin and the associated glycoprotein complex proteins are absent from the sarcolemmal membrane, resulting in altered mechanical and signaling functions and subsequently leading to immune cell infiltration, progressive muscle wasting, necrosis, and membrane fragility [1]. Indeed, the infiltration of inflammatory cells into the skeletal muscle is a notable characteristic of disease pathophysiology. Moreover, recent reports have demonstrated that inflammatory responses disrupt muscle homeostasis and inhibit processes that promote muscle repair and regeneration and that cytokines and chemokines produced by these inflammatory cells regulate the skeletal muscle inflammation observed in muscular dystrophy [2–5]. These findings therefore indicate that the degree of inflammatory cell infiltration is strongly associated with disease severity in muscular dystrophy patients.

This review points out inflammatory predisposition and immune-mediated mechanisms that regulate the disease severity of muscular dystrophy. We also discuss how the application of this knowledge could lead to novel therapeutic strategies.

### Characteristics of the inflammation involved in muscle diseases

In muscle diseases associated with chronic inflammation, the infiltration of muscle tissues by a variety of activated immune cells is typically heavily dependent on the presence of multiple cytokines [6]. Key cellular sources of these cytokines include CD4^+^ and CD8^+^ T cells, dendritic cells, B cells, neutrophils, and macrophages of both the pro-inflammatory M1 and the tissue regeneration-focused M2 phenotype. In particular, CD8^+^ T cells trigger muscle fiber death, and CD4^+^ T cells contribute to this process by providing inflammatory cytokines to CD8^+^ T cells and other immune cells [7, 8]. Meanwhile, macrophages perform a variety of important immunoregulatory and inflammatory functions and lyse muscle fibers through the production of nitric oxide, resulting in the release of high concentrations of cytolytic and cytotoxic molecules [9–11]. The high levels of tumor necrosis factor (TNF), interferon (IFN)-γ, and interleukin (IL)-12 observed in the blood and muscle tissues of patients with various types of myositis have implicated the T helper type 1 (Th1) response as a key mediator of the pathogenesis of these diseases [12]. Necrotizing myofibers are attacked by inflammatory cells at the endomysial, perimysial, and perivascular areas. Furthermore, a number of cytokines, including IL-1α and IL-17, can exert direct effects on the muscle tissue [13, 14] via the activation of signaling pathways, such as the nuclear factor NF-kB pathway, which further enhances the inflammatory response through up-regulation of cytokine/chemokine production. Notably, depending on their concentrations, these cytokines also display anti-inflammatory properties and exhibit duality of function [15]. Satellite cell-mediated regeneration is also mediated by several cytokines, as well as myofiber degeneration [16]. Thus, a tightly regulated, transient inflammatory response is required for normal muscle regeneration. The satellite cells which are muscle-resident stem cells get activated and start to proliferate as myoblasts upon muscle injury, then fuse and differentiate into myotubes that later grow, replacing damaged muscle [17, 18]. Dysregulated expression of cytokines such as TNF-α, TGF-β, or Il-1β leads to aberrant repair by chronic inflammation [19]. The prolonged inflammation is observed in severe myopathies such as Duchenne muscular dystrophy [20].

### Muscular dystrophy

Duchenne muscular dystrophy (DMD) is a severe X-linked muscular disease characterized by mutations in the gene encoding the cytoskeletal protein dystrophin that result in chronic inflammation, fibrosis, fat infiltration, and impaired vasoregulation, which manifests as muscle weakness and eventually leads to skeletal and cardiac muscle atrophy [21, 22]. Although mechanical injury and membrane defects are crucial factors that promote dystrophic disease pathology [23, 24], inflammation plays a large role in the muscle pathology of DMD. As an anti-inflammatory therapy, glucocorticoids, specifically prednisone and deflazacort, are widely used to improve muscle strength in DMD patients [25–27]; however, the beneficial effects of this therapy vary from patient to patient, and administration of these compounds sometimes result in negative side effects. Furthermore, it is currently unclear whether the efficacy of glucocorticoid therapy is dependent on the anti-inflammatory activity of these compounds, as glucocorticoids might also act directly on muscle fibers by stabilizing the sarcolemma [28, 29]. To develop new and improved therapeutic approaches for the treatment of DMD, it is essential to characterize the effects of chronic inflammation on disease progression.

### Inflammation in muscular dystrophy

In the pathogenesis of DMD, large numbers of inflammatory cells influence muscle pathology [7, 9, 30] [31–33]. However, the formation of muscle lesions is associated with immune cell infiltration that is clearly distinct from that which occurs during inflammatory responses to muscle injury in DMD patients and mouse models of muscular dystrophy [7, 9, 31, 32, 34]. The infiltrating mononuclear cells in the muscle tissues of DMD patients between 2 and 8 years of age are predominantly comprised of macrophages and T cells, while B cell infiltration is minimal [35, 36]. Meanwhile, in the muscles of *mdx* mice, the most widely used animal model for pathological analysis and evaluation of therapeutic approaches for DMD, the largest numbers of infiltrating immune cells were observed at 2–4 weeks of age. This infiltration, which was comprised of macrophages, T cells, and neutrophils, subsequently decreased in severity by 3 months of age [33].

Previous studies have observed the expression of pro-inflammatory factors (e.g., TNF-α, IFN-γ, IL-1, TGF-β, and MCP-1) prior to the onset of muscle degeneration in both DMD patients and *mdx* mice [37–39]. These factors also damage signals that have a profound impact on satellite cell behavior during the repair process. In an inflamed muscle of DMD, a persistently altered and reorganizing extracellular matrix (ECM) promotes damage and dysfunction. Exacerbated deposition of fibrin within the ECM promotes inflammation-mediated muscle degeneration and regeneration via α_M_β_2_ integrin engagement on macrophages, which eventually could lead to fibrosis development and loss of normal muscle architecture [18, 40]. M1 macrophages induce the expression of pro-inflammatory cytokines, IL-1β, TNF-α, and IL-6, which in turn may negatively regulate satellite cell functions [18].

Notably, depleting or inhibiting the expression of pro-inflammatory factors has resulted in significant improvements in dystrophic muscle pathology [30, 31, 41]. For example, TNF-α-deficient *mdx* mice exhibited improved pathological progression within the diaphragm and limb muscles compared to those of *mdx* mice expressing TNF-α [38]. IFN-γ expression is elevated in *mdx* muscles during the stage of the disease when macrophage-mediated muscle damage is rampant and numbers of M1 macrophages are greatly elevated [10]. Ablation of IFN-γ reduced muscle damage in *mdx* mice, showing the significantly lower pathological markers such as macrophage/neutrophil infiltration and necrosis of myofibers [42].

As a result, these pro-inflammatory cytokines are considered key factors in mediating the muscle damage caused by M1 macrophages [37]. In addition, the expression level of IL-10, which plays a particularly important role in mediating the switch from the M1 to the M2 phenotype through suppression of pro-inflammatory responses in dystrophic muscles, was observed to increase concurrently with those of TNF-α and IFN-γ during the acute stage of DMD (8- to 15-fold higher compared to that observed in wild-type muscles), thereby promoting muscle repair [10, 42].

### Immune-mediated regulation in DMD pathogenesis

IL-10 prevents the production of Th1-associated cytokines such as IFN-γ, TNF-α, IL-1β, and IL-6 in inflamed tissues [43]. As such, even low levels of IL-10 expression might affect the severity of inflammatory diseases and the immunopathology that results from high concentrations of pro-inflammatory cytokines. Indeed, IL-10-deficient mice display several features of the inflammatory bowel disease and Crohn’s disease [44], as well as increased susceptibility to *Helicobacter hepaticus*-induced colitis [45]. Moreover, IL-10 null mice did not exhibited severely reduced muscle strength due to severe inflammation [46], while older IL-10-deficient *mdx* mice presented with abnormal cardiac function that shared several characteristics with DMD-associated cardiomyopathy [47–49]. Notably, this change in cardiac function is paralleled by an increase in myocardial fibrosis and the occurrence of foci of myocardial necrosis and inflammation [50–53]. However, it remains unclear whether inflammation in the dystrophic muscle affects cardiac and respiratory dysfunction.

To study the effects of inflammatory predisposition on the severity of DMD, we previously generated mice lacking both dystrophin and IL-10 (*IL-10* ^*−/−*^ */mdx* mice) and subsequently demonstrated that these mice exhibit a phenotype that closely approximates that of DMD, as characterized by progressive muscle dysfunction associated with severe inflammation [54]. Indeed, compared to *mdx* mice, *IL-10* ^*−/−*^ */mdx* mice exhibited severe cardiac muscle degeneration and extensive myofiber loss with increased immune cell infiltration. Specifically, higher levels of CD68^+^ macrophage infiltration were detected in the diaphragms and heart muscles of *IL-10* ^*−/−*^ */mdx* mice than in those of *mdx* mice. Moreover, the cellular infiltration observed in *IL-10* ^*−/−*^ */mdx* mice increased with age, without the alternative activation of M2 (CD207^+^) phenotypes observed in *mdx* mice. We also showed that ablation of IL-10 in dystrophic muscles results in increased levels of macrophage infiltration and continuously distributed activation of M1 phenotype.

We detected increased levels of IL-1α, IL-1β, IL-1ra, IL-16, RANTES, M-CSF, MIG, JE/MCP-1, and TIMP-1 in the diaphragm and/or heart tissues of aged *IL-10* ^*−/−*^ */mdx* mice. NF-kB activity is thought to contribute to this up-regulation of pro-inflammatory factors, as the activity of this transcription factor is inhibited by IL-10 but not by other factors such as IL-6 and AP-1, or even by NF-kB itself [55]. Meanwhile, similar to IL-1 and the IL-1 receptor, increased production of IL-16, a potent chemoattractant for several immune cells, including monocytes and CD4^+^T cells [56], likely promotes further immune cell infiltration and activation within damaged muscles. Since IL-1β and IL-1ra are produced by M1 macrophages, our results suggest that there is strong activation of M1 macrophages in IL-10-deficient dystrophic muscles. We also demonstrated that elevated TGF-β signaling and type-I collagen expression resulted in widespread fibrosis within the diaphragm and heart tissues of *IL-10* ^*−/−*^ */mdx* mice but not in those of *mdx* mice.

**Fig. 1:**
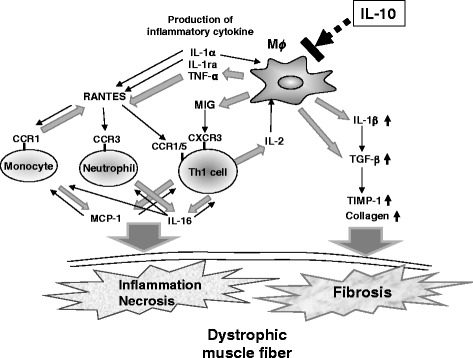
Function of interleukin (IL)-10 as an important immunomodulator that could regulate Duchenne muscular dystrophy (DMD) pathogenesis. In a dystrophic muscle, the inflammatory macrophage producing IL-1α, IL-1β, IL-1ra, IL-16, RANTES, M-CSF, MIG, JE/MCP-1, and TIMP-1 is regulated by IL-10. IL-10 might be an important immune-modulator in dystrophic muscles, because IL-10 ablation in *mdx* mice causes an increase in inflammation, muscle necrosis, and fibrosis

*IL-10* ^*−/−*^ */mdx* mice had a smaller average body mass at the juvenile stage and a significantly shorter life span than *mdx* mice; however, the diaphragms of both *mdx* and *IL-10* ^*−/−*^/*mdx* mice showed severe muscle degeneration and extensive myofiber loss with cell infiltration. Transient apnea was sporadically detected in *IL-10* ^*−/−*^/*mdx* mice, and juvenile *IL-10* ^*−/−*^/*mdx* mice displayed similar levels of fibrosis as aged *mdx* mice (8 months old). In association with the development of age-dependent heart failure, there was significantly increased cardiac fibrosis in the *IL-10* ^*−/−*^/*mdx* compared to the aged *mdx* mice. Lastly, echocardiographic analysis detected decreased fractional shortening (FS) percentage and ejection fraction percent (EF%) values in *IL-10* ^*−/−*^ */mdx* mice, indicating decreased left ventricular function with left and right ventricular dilatation (Fig. 1).

In summary, via pathophysiological analysis of *IL-10* ^*−/−*^ */mdx* mice, we confirmed that a predisposition to inflammation results in both chronic inflammation and more severe cardiorespiratory dysfunction. As such, these mice should comprise a useful DMD model for long-term observation of disease phenotypes under various therapeutic conditions.

### Therapeutic options

A number of anti-inflammatory therapies have been reported to have beneficial effects on DMD phenotypes [37]. For example, several anti-cytokine drugs that are currently available for use in human patients are capable of improving dystrophic muscle pathology [30, 57]. Meanwhile, the TNF-α blockers infliximab and etanercept and the IL-1 receptor antagonist anakinra, which have already been used for treatment of patients with rheumatoid arthritis and/or other inflammatory diseases [58, 59], could potentially be utilized in *mdx* mice [30, 57] and DMD patients. The proteasome inhibitor bortezomib has been shown to block NF-kB activation, thereby improving the appearance of DMD dog (GRMD) muscle fibers and reducing both connective tissue deposition and inflammatory cell infiltration [60]. Moreover, treatment with an adeno-associated virus vector encoding a short hairpin RNA (shRNA) that specifically targets NF-kB ameliorated muscle pathologies in *mdx* mice [61].

The clinical interest in the therapeutic application of mesenchymal stromal cells (MSCs) is based on the anti-inflammatory properties of these cells and their ability to release cytokines into the surrounding environment, thereby modifying the developmental fate of neighboring cells. Combinatorial application of the immunosuppressive and/or anti-inflammatory effects and myogenic differentiation of MSCs comprises a promising therapeutic approach for treating muscle diseases.

## Conclusions

In this review, we introduced immune-mediated systems that regulate the time course of disease progression in muscular dystrophy. We also suggested that IL-10 comprises an important immunomodulator that could be utilized to regulate the pathogenesis of this disease. Indeed, IL-10-based strategies would be promising for the treatment of cardiac and respiratory dysfunction in DMD. These findings are important for the development of effective therapies using anti-inflammatory drugs and/or immunomodulatory stem cells, such as MSCs, to improve muscle and cardiorespiratory dysfunction.

## Abbreviations

DMD, Duchenne muscular dystrophy; IFN, interferon; IL, interleukin; MHC, myosin heavy chain; MSCs, mesenchymal stem cells; Th1, T helper type 1; TNF, tumor necrosis factor
